# Automatic identification of coronary tree anatomy in coronary computed tomography angiography

**DOI:** 10.1007/s10554-017-1169-0

**Published:** 2017-06-24

**Authors:** Qing Cao, Alexander Broersen, Michiel A. de Graaf, Pieter H. Kitslaar, Guanyu Yang, Arthur J. Scholte, Boudewijn P. F. Lelieveldt, Johan H. C. Reiber, Jouke Dijkstra

**Affiliations:** 1 Division of Image Processing, Department of Radiology, C2S, Leiden University Medical Center, PO Box 9600, Albinusdreef 2, 2300 RC Leiden, The Netherlands; 2 Department of Cardiology, Leiden University Medical Center, Leiden, The Netherlands; 3 Medis Medical Imaging Systems BV, Leiden, The Netherlands; 4 Laboratory of Image Science and Technology, Southeast University, Nanjing, China

**Keywords:** Coronary computed tomography angiography (CCTA), Coronary artery labeling, Coronary artery dominance

## Abstract

An automatic coronary artery tree labeling algorithm is described to identify the anatomical segments of the extracted centerlines from coronary computed tomography angiography (CCTA) images. This method will facilitate the automatic lesion reporting and risk stratification of cardiovascular disease. Three-dimensional (3D) models for both right dominant (RD) and left dominant (LD) coronary circulations were built. All labels in the model were matched with their possible candidates in the extracted tree to find the optimal labeling result. In total, 83 CCTA datasets with 1149 segments were included in the testing of the algorithm. The results of the automatic labeling were compared with those by two experts. In all cases, the proximal parts of main branches including LM were labeled correctly. The automatic labeling algorithm was able to identify and assign labels to 89.2% RD and 83.6% LD coronary tree segments in comparison with the agreements of the two experts (97.6% RD, 87.6% LD). The average precision of start and end points of segments was 92.0% for RD and 90.7% for LD in comparison with the manual identification by two experts while average differences in experts is 1.0% in RD and 2.2% in LD cases. All cases got similar clinical risk scores as the two experts. The presented fully automatic labeling algorithm can identify and assign labels to the extracted coronary centerlines for both RD and LD circulations.

## Introduction

As a non-invasive imaging modality, coronary computed tomography angiography (CCTA) is widely used for the diagnosis of cardiovascular disease [1]. It provides detailed information about the anatomy of the coronary arteries and the characteristics of coronary atherosclerosis such as the extent of calcifications, the volumetric plaque burden, degree of stenosis and occlusions. In clinical practice, radiologists and cardiologists usually report these pathological findings per artery or per segment according to the society of cardiovascular computed tomography (SCCT) image guidelines [2] and CAD-RADS™ reporting system [3].

Previous studies have demonstrated the clinical significance of stenosis localization. For example, a different weight factor is applied to each coronary segment in the SYNTAX score [4] which is designed to determine the extent and complexity of coronary artery disease (CAD). A worse prognosis for patients with acute myocardial infarction is caused by a proximal located lesion compared to more distal located lesions [5, 6]. Also, previous studies have shown that automatic quantification of CCTA images is feasible [7, 8]. Therefore, automated lesion reporting and risk stratification requires an automatic coronary artery extraction and identification algorithm.

Identification of the coronary tree anatomy, i.e. automatically assigning labels to the segments of coronary trees was limited to the right dominant (RD) coronary trees in most previous studies [9–12]. Although ~86% of patients have a RD [13] coronary system, a widely applicable system should also be able to deal with left dominant (LD) coronary trees [14–16].

A number of previous methods have shown that the centerlines of coronary arteries in CCTA images can be extracted automatically [17–19]. This paper presents a labeling method to automatically identify and assign labels to the anatomical segments of the entire coronary tree. The assigned label and the location of the start and end points of the label are compared with the results from human observers. Furthermore, current clinical risk scores are computed to show the performance of the identification method in risk score assessment.

## Materials and methods

### Patients

The patient population consisted of 100 clinical datasets (62 RD cases and 38 LD cases), including: five RD cases to refine the RD model which was derived from Dodge et al. [20]; 11 LD cases to build and train the LD model; and the remaining 84 cases for testing and evaluation of the method. The 100 datasets did not include cases with severe lesions at the proximal parts of the main branches or coronary anomalies. The institutional review board of the Leiden University Medical Center approved this retrospective evaluation of clinically collected data. The need for written informed patient consent was waived.

The labeling method was applied to the extracted centerlines of the coronary trees. Cases with heavily calcified plaques or step/motion artifacts were handled similarly as long as the centerlines were successfully extracted or manually corrected by experts. The coronary centerlines for all the 100 datasets were extracted by a method presented by Yang et al. [18].

### CTA acquisition

Data acquisitions were performed with a 64-detector row CT scanner (Aquilion 64, Toshiba Medical Systems, Tokyo, Japan) or 320-detector row CT scanner (Aquilion One, Toshiba Medical Systems, Tokyo, Japan) according to a previous described protocol [21]. In short, if the heart rate was higher than 65 beats per minute, oral or intravenous β blockers were administered, if not contra-indicated. In total, 60–110 mL non-ionic contrast material (Iomeron 400, Bracco, Milan, Italy or Ultravist 370, Bayer Schering Pharma AG Berlin, Germany) was administered followed by a saline flush with a flow rate of 5 mL/second. Thereafter, images were reconstructed at the best phase of the R–R interval. The average image size and voxel size of the datasets were 512 × 512 × 512 and 0.307 × 0.307 × 0.25 mm, respectively.

### Automatic tree labeling method

**Fig. 1:**
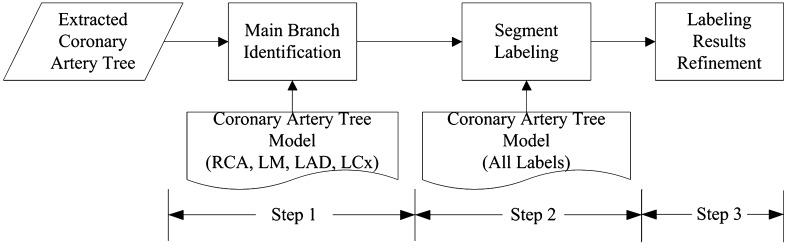
Different steps in the identification of all the segments in the coronary artery tree

Figure 1 displays different steps in the identification of all the segments in the coronary artery tree. A three-dimensional (3D) coronary tree model provides anatomical a priori knowledge of coronary arteries. With the 3D model, a three-step labeling method is used to perform the identification: (1) Align the model with the patient coronary tree to identify the main branches, and separate the coronary tree into sub-trees according to the main branches; (2) Evaluate the matching costs for the segments in each sub-tree to find optimal correspondence between model and patient tree; and (3) Apply logical rules which were translated from the clinical experience to adjust and refine the labels on all segments to obtain the final labeling.

#### Coronary artery tree model

Both RD and LD models are composed of three main branches: right coronary artery (RCA), left anterior descending (LAD), left circumflex (LCX), and their derived side-branches. The labels in the models are based on the 15-segments model defined by the American Heart Association (AHA) [22], which is widely adopted in the clinical practice. Additionally, ramus intermedius (RI) arteries which originate near the LAD-LCX bifurcation are added to the models. Furthermore, the obtuse marginal (OM) branches are distinguished as the first obtuse marginal (OM1) and the second marginal (OM2) according to the SCCT guidelines [2]. All the labels used in this paper are summarized in Table 1.

**Table 1:** Labels used in the coronary artery tree labeling

	Main branch	Labels of main branch	Labels of side-branches
		LM	/
Sub-trees	RCA	pRCA, mRCA, dRCA	Right posterior lateral (RPLB) branch RD type: right posterior descending (RPDA) artery
	LAD	pLAD, mLAD, dLAD	Two diagonal arteries (D1, D2)
	LCX	pLCx, LCx	Two obtuse marginal (OM1, OM2) arteries, left posterior lateral (LPLB) branch Anterolateral (AL) artery or ramus intermedius (RI) arteries LD type: left posterior descending (LPDA) artery

*LM* left main artery, *RCA* right coronary artery, *LAD* left anterior descending, *LCX* left circumflex, *LD* left dominant, *RD* right dominant, *p* proximal, *m* mid, *d* distal

Right dominant model: The initial 3D RD model was created using the 2D angiography statistical information from Dodge et al. [20]. Then five randomly selected RD cases were used to refine and obtain the final RD model. The initial results of this RD model were presented by Yang et al. [12]. The balanced type cases were treated as RD in this paper.

Left dominant model: Coronary artery dominance is defined in terms of which artery supplies the posterior descending artery (PDA) [23]. Because of the difference in PDA and LD cases were not included in Dodge et al. [20], a separate LD model was created. The LD model was built from 11 randomly selected training-datasets using a leave-one-in cross validation scheme as follows. Each time, one of the 11 cases was chosen as the initial LD model, after which the lengths of the branches of the model were normalized to the average lengths of the 11 training datasets. The remaining ten training cases were used to validate the model. Finally, the model with the best validation results was defined to be the final LD model. Additionally, the LPDA was defined as the end of the LCX. The distal part (dRCA) of the RCA was excluded from the LD model, since the dRCA was not present in the selected training datasets. From a clinical point of view, the discrimination of proximal (p-), mid (m-) or distal (d-) RCA segment is not important in LD cases.

#### Main branch identification

The patient coronary tree has a different location, orientation and size compared to the model, so a point-set registration method [24] is introduced to align the 3D model with it. Before the alignment, centerlines of the patient coronary tree and the model are normalized and re-sampled to remove scale variance; all side-branches from the 3D model are removed to reduce their influence on the registration. Weight factors, defined as the number of all child arteries originating from the current segment, are assigned to the points in the patient coronary tree to ensure that their main branches attract the main branches in the model.

RCA, LAD, or LCX is identified as the centerline in the patient coronary tree with the minimal distance to the corresponding main branch in the aligned model. The overlapping part of the identified LAD and LCX is marked as LM. Branches derived from the LAD–LCX bifurcation are labeled as RI arteries. This step provides an initial identification of the main branches in the patient coronary tree, because the distal parts of the main branches have a lower weight factor as their side-branches. In the next section, an iterative algorithm is described to find the optimal correspondence of each label in the 3D model.

#### Segment labeling

Before labeling all the segments, short side-branches (less than 1 cm) and side-branches that have obtuse angles (more than 120° away from the main branches) at the bifurcations are removed. The rigid transformation obtained in the previous step is used to deform the 3D model with all side-branches.

**Fig. 2:**
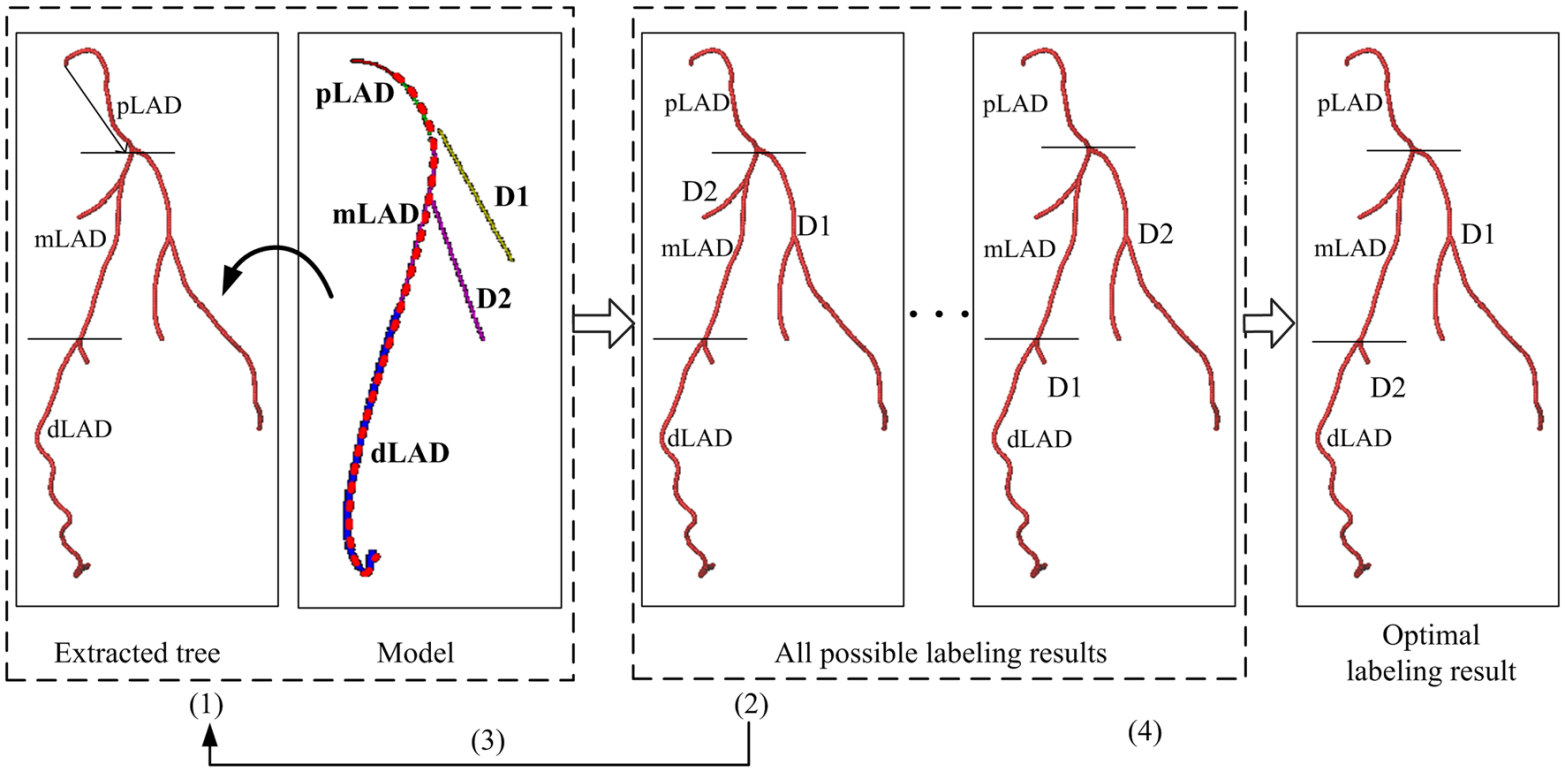
The iterative algorithm for labeling all segments. Abbreviations can be found in Table 1

By minimizing a cost function, an iterative algorithm is applied to find the optimal labeling result from all possible labeling results [12]. According to the identified three main branches, the extracted coronary tree can be separated into three sub-trees with each sub-tree containing one identified main branch and several side-branches. Figure 2 illustrates the iterative process of labeling one of the sub-trees. Three sub-trees are subsequently matched with the corresponding sub-trees in the model to get all the segments labeled.

#### Labeling results refinement

In clinical practice, the proximal, mid and distal parts of LAD and LCX are separated at the bifurcations of the specific side-branches according to AHA coronary artery classification [22]. The labeling result obtained from the previous two steps as shown in Fig. 2 may not satisfy these requirements. Some criteria were defined in Yang et al. [12] to adjust the obtained labeling result.

Additionally, by transforming clinical experience into logic rules, RI branches are discriminated into RI and anterolateral (AL) branches based on the distance of their origin from the LAD–LCX bifurcation. The distance threshold for RI branches is defined as 0.5 cm according to the clinical experience of cardiologists. If the side-branches originate from the LCX and the distance from its opening to the LAD–LCX bifurcation is less than 2 cm, these side-branches are labeled as AL branches. Branches bifurcating after more than 2 cm from the LCX ostium are treated as OM branches.

If these cases mentioned above are not present in the labeling result, the initial labeling result will not be changed.

### Evaluation measures

For each label, the presence and the accuracy of the start and end points are evaluated. In order to validate the clinical performance of the identification method on each patient or each coronary tree in the aspect of risk score assessment, the accuracy for clinical risk scores is also calculated. Automatic labeling results were compared with the manual labeling from two experts. Two experts with at least 4 years of experience in cardiac CT imaging independently assigned labels to the coronary tree segments, and subsequently verified the results for each other to correct any mistakes. As differences between the experts remained after their verification, inter-observer variability of the manual labeling is also analyzed.

#### Presence

In this step, evaluate the labels in Table 1 present or not. As the automatic method may omit or wrongly assign the label on some segments and different opinions also exist between the two experts, three situations are considered. For each label: (1) If both experts agreed with the result of the automatic method, the automatic assigned label is treated as definitely correct; (2) If both experts disagreed with the results of automatic method, automatic assigned label is treated as definitely wrong; (3) If expert1 disagreed with expert2, this means either of them would agree with the automatic method. In this situation, the presence of the label is ambiguous, automatic assigned label is treated as a semi-correct.

#### Overlap

**Fig. 3:**
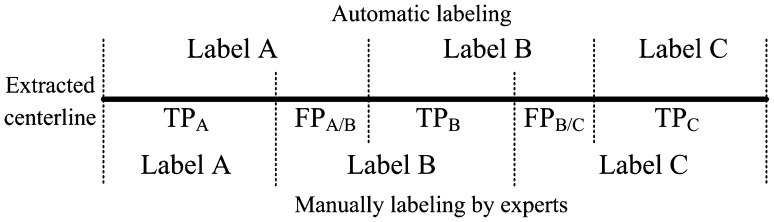
Definition of the overlap measure used in the evaluation. *TP* true positive, *FP* false positive

After the evaluation of the presence of labels, the start and end points of a labeled segment are compared with the results of the two experts. An overlap measurement is defined in Fig. 3 to quantify the labeling accuracy for each presented label. Along the extracted centerline, the points with the same label in both automatic and experts labeling results are marked as true positive (TP), otherwise (i.e. longer or shorter part of the segments), marked as false positive (FP). The overlap measure for the label A between automatic and experts labeling results is defined as.

$${\text{O}}{{\text{V}}_{\text{A}}}=\frac{{\left\| {{\text{T}}{{\text{P}}_{\text{A}}}} \right\|}}{{\left\| {{\text{T}}{{\text{P}}_{\text{A}}}} \right\|+\left\| {{\text{F}}{{\text{P}}_{\text{A}}}} \right\|}}$$

#### Accuracy for clinical risk scores

The labeling accuracy for current clinical risk scoring models is evaluated for all patients. In current literature, several risk scores of the coronary plaques in the coronary tree are available to provide an estimate of prognosis of cardiovascular diseases [25, 26].

In order to fit the clinical meaning, assume that all the segments have plaques in this method, four scores are calculated. (1) The ability to identify all segments of the three main branches (i.e. RCA, LAD, LCX). (2) The ability to label the proximal segments of these three main branches including LM. (3) The segment involvement score (SIS) [27] per patient is calculated. SIS is defined as the total number of correctly labeled segments with regard to the segments labeled by experts. (4) The Leaman score [25], as also applied in the SYNTAX-Score [4], is computed by assigning a weight factor ranging from 6 (LM in LD) to 0 (p-, m-, d-RCA in LD) to each coronary segment. For each patient, the Leaman scores are calculated as the summation of the weight factors of all the correctly labeled segments. The labeling of OM in the Leaman score is correct if one of the OM1 or OM2 is labeled correctly. Both SIS and the Leaman score are shown as the proportion compared to the results of experts.

#### Statistical analysis

The presence of each label is reported as an absolute number. Agreements or disagreements of the presence are expressed as percentages. The accuracy for overlap and clinical risk scores are illustrated as absolute numbers or percentages ± standard deviation (SD) where appropriate.

## Results

**Fig. 4:**
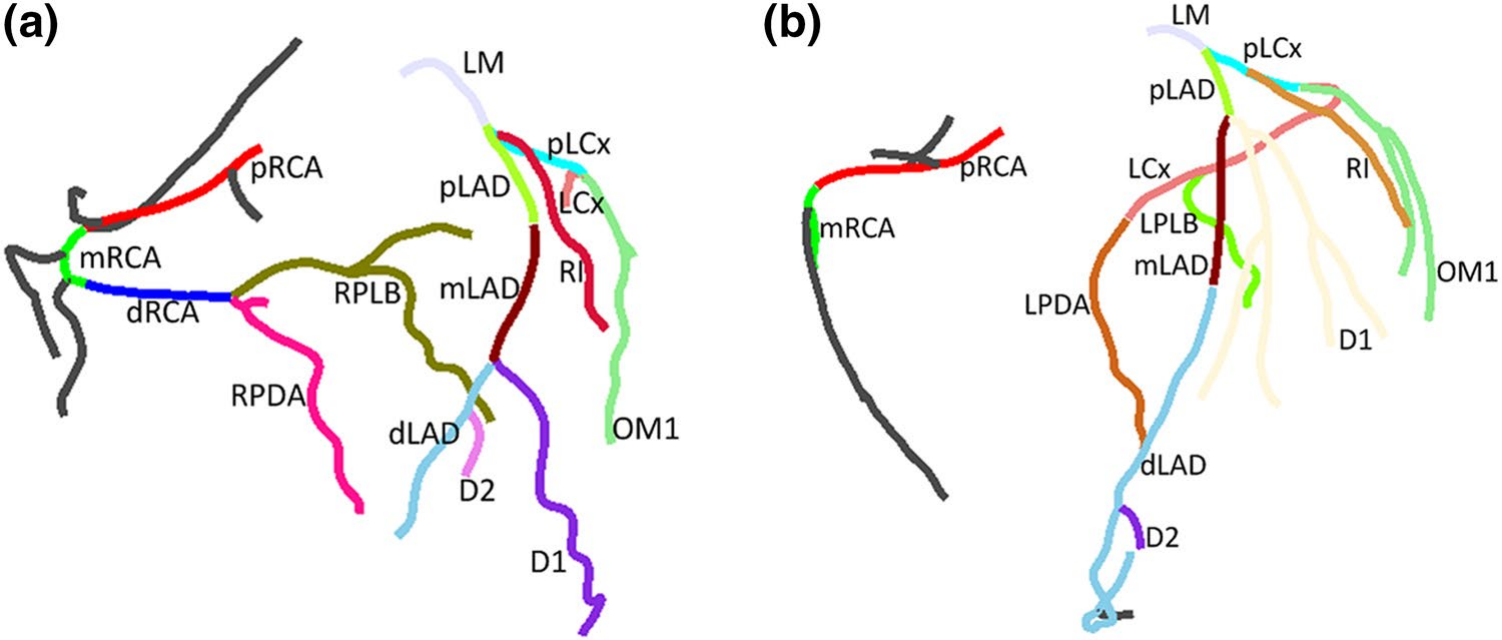
Coronary artery tree labeling result with their labels surrounded by **a** RD coronary tree, **b** LD coronary tree. All segments including proximal, mid and distal parts as well as side-branches were labeled correctly

The automatic labeling of one coronary tree took less than 3 s on a PC with a Quad Core 2.4Ghz processor and 8 GB RAM. One dataset was excluded from the RD evaluation datasets because of an extraction problem of LAD. In total, 83 (56 RD and 27 LD) cases were used in the evaluation. Baseline characteristics of the 83 patients are depicted in Table 2. 61 patients were male and the mean age was 59.9. A total number of 1149 (795 for the RD cases and 354 for the LD) segments were included on which the automatic method or experts assigned any labels. Figure 4 shows the results of applying the automatic labeling approach on a RD and a LD case.

**Table 2:** Patient characteristics

	Total (83)
Age (years)	59.9 ± 11.4
Gender (% male)	61 (73%)
Diabetes	20 (24%)
Hypertension^a^	34 (41%)
Hypercholesterolemia^b^	37 (45%)
Family history of CAD*	26 (31%)
Smoking	15 (18%)
Obesity	22 (27%)

Data are represented as mean ± SD or as number and percentages of patients

*CAD* coronary artery disease

*Defined as the presence of coronary artery disease in first-degree family members at age <55 years in men and <65 years in women

^a^Defined as systolic blood pressure ≥140 mm Hg and/or diastolic blood pressure ≥90 mmHg or the use of antihypertensive medication

^b^Defined as serum total cholesterol ≥230 mg/dL or serum triglycerides ≥200 mg/dL or treatment with lipid lowering medication

**Fig. 5:**
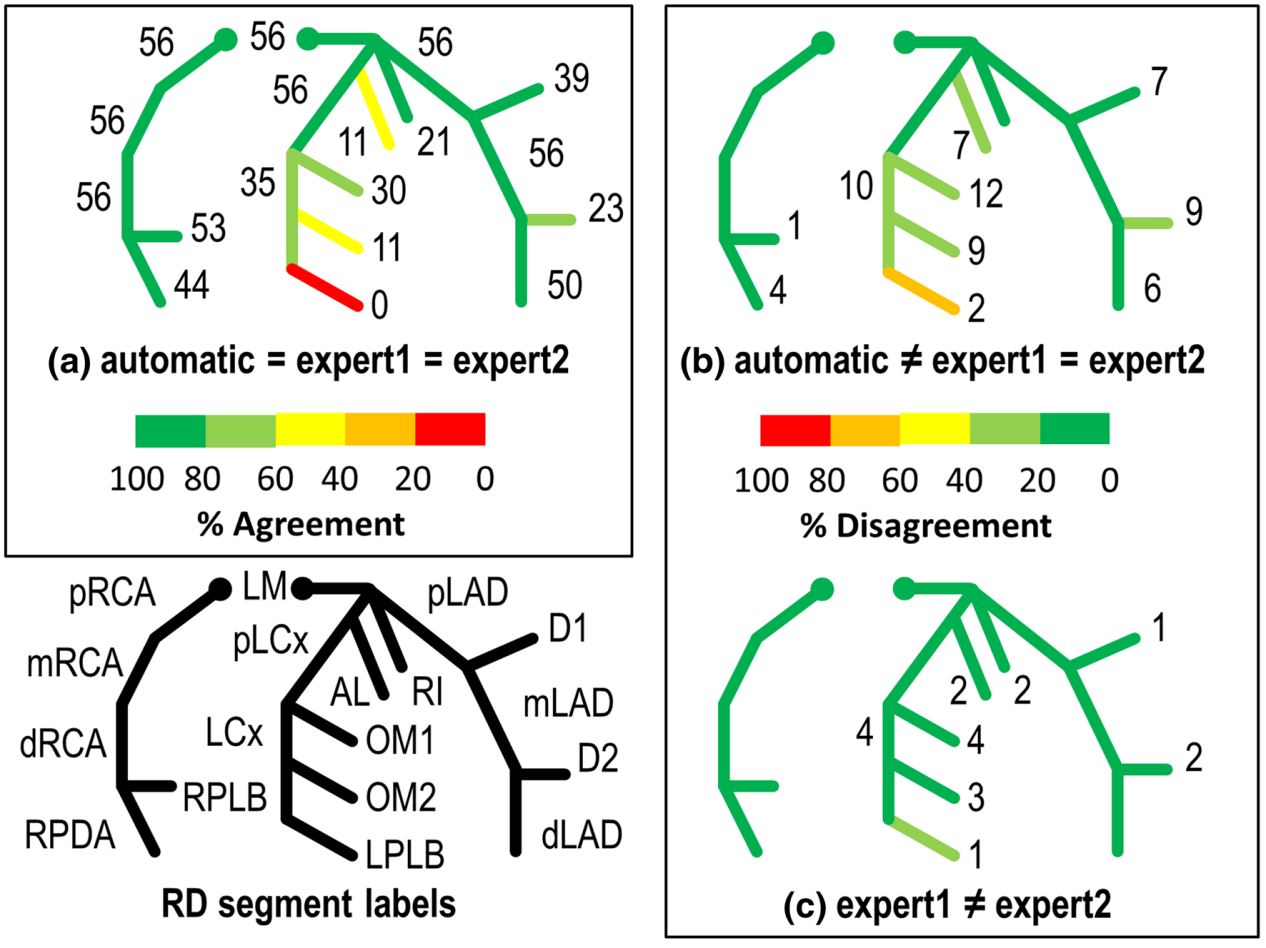
Agreements and disagreements among expert1, expert2 and the automatic method for RD cases. **a** Agreements among expert1, expert2 and automatic method, **b** disagreements between experts and automatic method, **c** disagreements between expert1 and expert2. For each label, numbers show the amount of agreed or disagreed segments, and by adding all the numbers in (**a**), (**b**) and (**c**) can get the total numbers of the segments involved. The percentages of agreement and disagreement in comparison with the total segments involved are illustrated in *different colors*, and *green color* shows a 100.0% agreement (**a**) or 0.0% disagreement (**b**) and (**c**). For instance, 0 (100.0%) out of total three LPLB segments get agreements among both experts and automatic method in (**a**) while in 2 (66.6%) LPLB segments, both experts disagreed with automatic method in (**b**) and in 1 (33.3%) LPLB segment that expert1 didn’t agree with expert2 in (**c**). Abbreviations can be found in Table 1

**Fig. 6:**
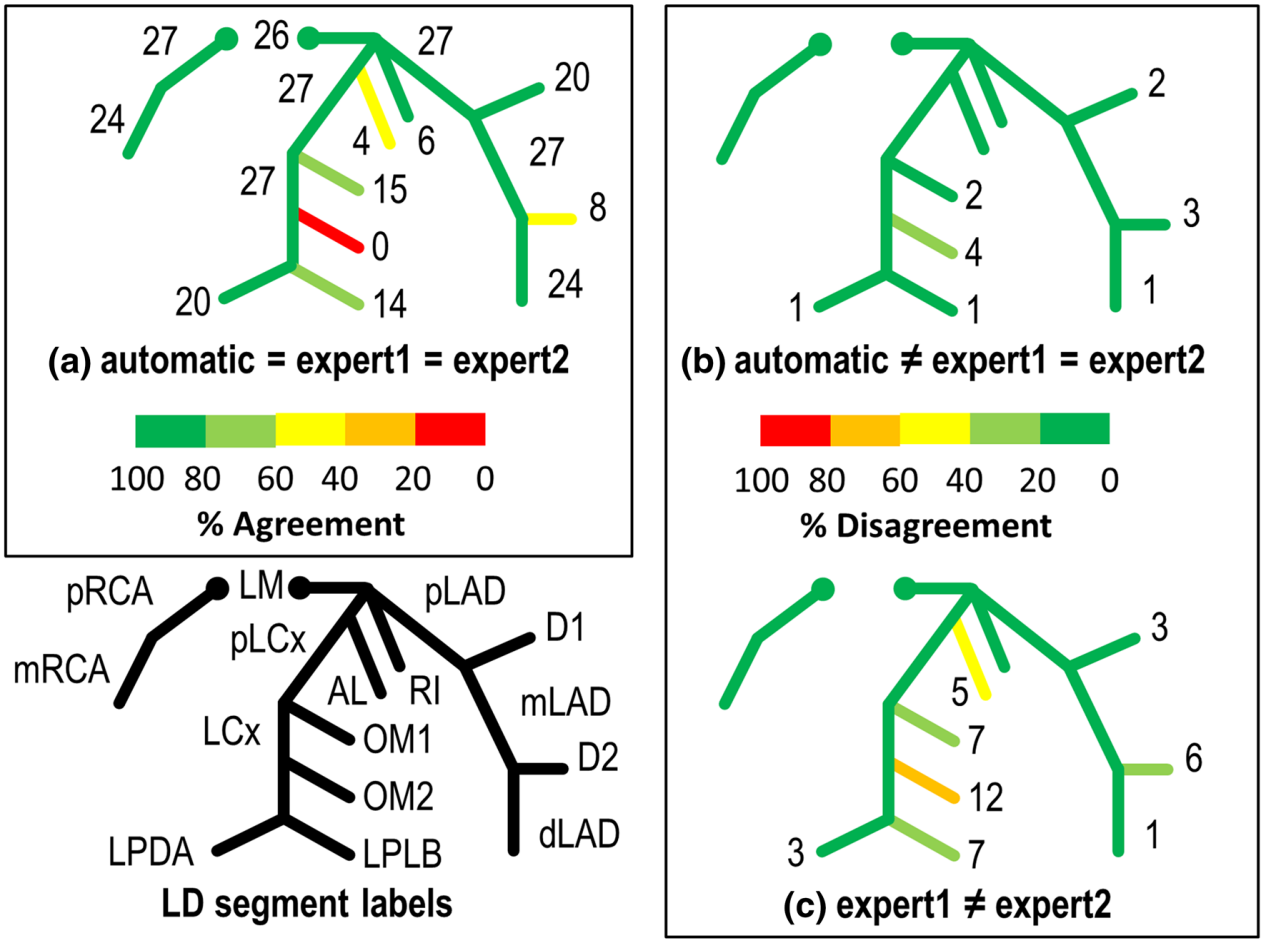
Agreements and disagreements among expert1, expert2 and automatic method for LD cases. **a** agreements among expert1, expert2 and automatic method, **b** disagreements between experts and automatic method, **c** disagreements between expert1 and expert2. For each label, numbers show the amount of agreed or disagreed segments, and by adding all the numbers in (**a**), (**b**) and (**c**) can get the total numbers of the segments involved. The percentages of agreement and disagreement in comparison with the total segments involved are illustrated in *different colors*, and *green color* shows a 100.0% agreement in (**a**) or 0.0% disagreement in (**b**) and (**c**). Abbreviations can be found in Table 1

Figures 5 and 6 show an overview of agreements and disagreements on the presence of labels among the automatic method, expert1 and expert2 for RD and LD coronary trees, respectively. For each label, numbers show the amount of agreed or disagreed segments, and by adding all the numbers in a, b and c, one can get the total numbers of the segments involved. For instance, in the RD datasets (Fig. 5) the total number of segments with label OM2 can be computed as follows. Starting with 11 segments in which all agree from Fig. 5a plus 9 where the experts disagree with the automatic from Fig. 5b, and plus 3 where both experts disagree from Fig. 5c makes a total of 23 segments. The percentages of these agreements and disagreements with respect to the total segments involved are illustrated in different colors, and green color shows a 100.0% agreement (Fig. 5a) or 0.0% disagreement (Fig. 5b, c).

### Presence

The agreement on the labeling of the two experts is 776 (97.6%) RD segments and 310 (87.6%) LD segments by adding the numbers in Figs. 5a, b and 6 a, b, respectively. Seen from Figs. 5a and 6a, the presence of the labels on 709 (89.2%) RD and 296 (83.6%) LD segments were definitely correct, on which both experts and the automatic approach assigned the same labels. For all the cases, the labels of the proximal segments (pRCA, pLAD and pLCx) including LM were always present and got a 100.0% agreement.

Figures 5b and 6b show the segments with definitely wrong labels where both experts disagreed with automatic method. However, only 67 (8.4%) RD and 14 (4.0%) LD segments had definitely wrong labels and most of the differences (RD 55.2%; LD 78.6%) were on diagonal and OM branches.

Figures 5c and 6c show the segments (91.6% in RD, and 96.0% in LD) with semi-correct labels where expert1 disagreed with expert2 but either of them agreed with automatically assigned labels. Expert1 disagreed with expert2 about the presence of the labels on 19 (2.4%) RD segments and 44 (12.4%) LD segments. Specifically, for segments with OM2 labels in the LD cases, expert1 disagreed with expert2 on 12 (75.0%) out of 16 segments.

In RD cases, 0 out of 3 segments with LPLB labels were definitely correct, while the disagreements between expert1 and expert2 were also 33.3%. In LD cases, no OM2 segments got definitely correct labels, and 4 (25.0%) got definitely wrong labels, while on the remaining 12 (75.0%) OM2 segments, either expert1 or expert2 agreed with the automatic method.

### Overlap

**Fig. 7:**
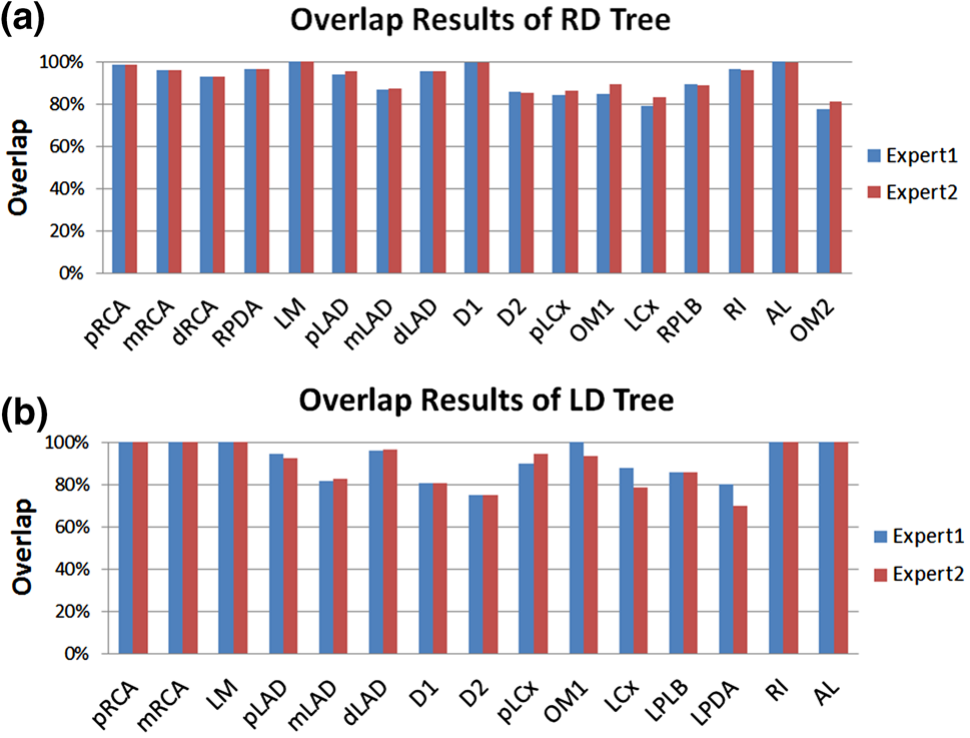
Overall overlap results of the methods. **a** and **b** show the overlap of each segment with two experts for RD tree and LD tree, respectively. *RD* right dominant, *LD* left dominant; The other abbreviations used here are the same as in Table 1

The average overlap accuracy of the definitely correct labeled segments (as shown in Figs. 5a, 6a) is depicted in Fig. 7. All labels got at least 70.0% overlap and the LM even has a 100.0% overlap. 18 labeled RD segments and 7 LD segments have no overlapping regions which appeared more often in certain segments, such as D2 (3 in RD, 2 in LD), LCx (4 in RD), and the posterior branch (4 RPLB and 2 LPLB).

There are also inter-observer differences in the overlap on some labels as shown in Fig. 7, which is 1.0% in RD cases and 2.2% in LD cases in average. In both RD and LD cases, there was a larger inter-observer variability on the labels of LCX sub-tree segments, especially on LPDA segments of LD cases that there was an average 10.0% overlap difference.

By averaging the overlap differences between the experts, the average overlap accuracy on RD labeling is 92.0% (±6.7%), and on LD is 90.7% (±9.5%).

### Accuracy for clinical risk scores

Clinical risk scores of RD and LD cases are shown in Table 3 with the differences in experts averaged. The tree labeling method was able to accurately identify all the proximal segments and LM segments for all patients and at least one main branch was labeled correctly. From all clinical risk scores listed in Table 3, RD and LD coronary trees got similar labeling results.

**Table 3:** Clinical risk scores of RD and LD cases

	RD	LD
	Patients (n = 56) auto/expert (%)	Patients (n = 27) auto/expert (%)
Identify three main branches		
N = 1	100.0%	100.0%
N = 2	98.2%	98.1%
N = 3	70.5%	96.3%
Proximal segments and LM	100.0%	100.0%
SIS		
Mean(±SD)	92.6% (±7.3%)	93.3% (±8.1%)
=100.0%	37.5%	46.3%
≥90.0%	68.8%	70.4%
≥80.0%	98.2%	90.7%
<80.0%	1.8%	9.3%
Leaman score		
Mean(±SD)	96.0% (±3.8%)	96.7% (±4.1%)
=100.0%	37.5%	50%
≥95.0%	67.9%	70.4%
≥90.0%	95.5%	96.3%
<90.0%	4.5%	3.7%

Data are represented as percentages or mean ± SD. N represent the numbers of branches. The SIS and Leaman scores are shown as the percentages of automatic method results in comparison with the averaged results of experts. For example, there were 37.5% RD and 46.3% LD patients got the same SIS score as the experts

*RD* right dominant, *LD* left dominant, *LM* left main branch, *SIS* segment involvement score, *SD* standard deviation

All segments of the three main branches were identified in more than 70.5% of the RD cases and 96.3% of the LD cases and the wrongly labeled segments all occurred in the distal part (dLAD or dLCx). The SIS percentages are 92.6% (±7.3%, RD) and 93.3% (±8.1%, LD) in comparison with manual labeling results. Specifically, in only 1.8% of the RD patients and 9.3% of the LD patients, the SIS score is less than 80.0% compared to the experts. For the segments which have a weight >0 in the Leaman score system, the automatic method got at least 96.0% similar Leaman scores compared to the manual labeling results. In only 4.5% of the RD patients and 3.7% of the LD patients, the automatic method is less than 90.0% similar to the Leaman scores from the experts.

## Discussion

In this paper, an automatic coronary artery tree labeling algorithm for centerlines extracted from CCTA images is presented. The labeling algorithm can automatically identify coronary tree segments and assign labels to the identified segments for both RD and LD circulations. This can be used to facilitate automatic lesion reporting and risk stratification in a large cohort of patients [8] and allow automatic follow-up comparison of quantitative parameters on certain segments.

### Evaluation of the algorithm

#### Presence

The accuracy for the presence of D1, D2, OM1 and OM2 labels are lower compared to other segments, because labels of the D1 or D2 and OM1 or OM2 were often switched, especially when one of the diagonal branches or marginal branches did not exist or were absent from the extraction. More disagreements in experts on these segments show that it is also difficult for human experts to discriminate the D1 or D2 and also OM1 or OM2 segments. However, with regard to risk assessment, whether it is the D1 or D2 is not important as long as they are identified as diagonal branches. The same applies to the marginal branches.

#### Overlap

On LPDA segments of LD cases, there is a large overlap inter-observer variability (10.0%) which is caused by the different start point definition of LPDA. In this method, the start point of LPDA is defined at the position where LCx starts to go to the ventricle groove which is consistent with expert1, while expert2 used the bifurcation point of LCx and LPLB as a start point.

In general, the middle segment of a branch usually has a lower overlap score compared to the proximal segment of the same branch. Due to the definition of pLAD, a missing label of the D1 or the lack of extraction of the D1 will create an incorrect end point for the pLAD. Similarly, if the D2 and OM1 are not extracted, it will influence the accuracy of the mLAD and pLCx. Furthermore, if one of the segments was assigned the wrong label or wrong start and end points, the following segment will inherit or even enlarge this error.

#### Accuracy for clinical risk scores

The presented labeling method is capable to accurately identify all the proximal segments of main branches and can get similar results as the experts with respect to SIS and Leaman scores. Although, only 70.5% RD cases were labeled correctly in all three main branches compared to experts, the errors all occurred in distal parts of the main branches. It should be taken into account that the lesions in the distal parts have less clinical relevance than in the proximal parts [5, 6].

### Comparison to other labeling methods

Several approaches [9, 28] focused on the coronary tree labeling in 2D X-ray angiography. Since 2D X-ray angiography is a different imaging modality with CCTA, assigning the anatomical labels to coronary arteries in CCTA images has different challenges. To the best of our knowledge, the literature on automatic coronary tree labeling in CCTA images is very limited.

Akinyemi et al. [10] presented an automatic labeling method which used geometric features of coronary arteries to train a multivariate Gaussian classifier. In this method, the large anatomical variation of the training datasets such as the size of the heart might decrease the accuracy of the labeling results, while our method is robust to the scale of the coronary trees. The proximal, mid and distal parts of the main coronary arteries were not identified, which is widely adopted in clinical practice for CCTA image reporting and evaluating.

Recently, Mehmet et al. [11] proposed a coronary labeling method through calculating the geodesic paths between coronary tree of a standard model and the patient. In the method, labeling a whole coronary tree took 3 min by parallelized implementation, while we only need less than 3 s without parallelization. Anatomical prior location, such as the position of four chambers, was used to set the coordinates of the coronary tree, while only coronary centerline points were needed in our automatic method. Furthermore, their approach was not used on LD cases or on cases with a RI. A similar overlap measurement was used to evaluate the labeling accuracy. Compared to their labeling results (87.0% for left coronary tree and 86.0% for right coronary) on automatic detected centerlines, our method got a slightly higher labeling score (92.0%, RD). Since the datasets, the centerline detection methods and coronary segments model are different, it’s hard to put these results side-by-side.

### Limitations

The following limitations of the present study should be considered. First, two models for RD and LD cases are needed, thus the dominance type of the coronary tree should be known before labeling. Automatic detection of the dominancy to choose the correct model or building a generic model for all the three main dominant types will be investigated in future work. Second, the quality of the tree labeling is highly dependent on the automatic extraction results. In follow up work, we will study if the method could determine whether there are missing, shortened or wrongly extracted arteries. In this way, the labeling of the coronary arteries will allow to improve the tree extraction results by automatically extending short branches and remove veins from an extracted tree.

## Conclusion

The presented labeling algorithm can successfully identify the coronary tree anatomy in CCTA automatically for both RD and LD cases in a fully automatic manner.
